# Adaptation and psychometric properties of the Spanish Perceived Ageism Questionnaire (PAQ)

**DOI:** 10.3389/fpsyg.2026.1823220

**Published:** 2026-09-11

**Authors:** María del Sequeros Pedroso-Chaparro, Ariadna de la Vega Castelo, Carlos Vara-García, Elena Brenlla, Isabel Miguel

**Affiliations:** 1 Department of Psychology, Universidad a Distancia de Madrid, Madrid, Spain; 2 Department of Psychology, Universidad Rey Juan Carlos, Alcorcón, Spain; 3 Department of Psychology, Universidad Católica Argentina, Buenos Aires, Argentina; 4 RISE-Health (RISE-Health@UPT), Portucalense University, Porto, Portugal

**Keywords:** measurement invariance (MI), perceived ageism, psychometric properties, scale, Spanish version

## Abstract

**Objectives:**

Perceived ageism is a critical variable for understanding mental health and wellbeing across the lifespan. The Perceived Ageism Questionnaire (PAQ) assesses subjective experiences of both negative and positive perceived ageism through dimensions of stereotypes, prejudice and discrimination. The aim of this study was to (a) adapt the PAQ into Spanish and analyze its psychometric properties and (b) examine the associations between perceived ageism, mental health, self-perceptions of aging and age.

**Method:**

A sample of 404 adults living in Spain (aged 18 to 95 years old) completed the Spanish version of the PAQ along with measures of depression, anxiety, stress, life satisfaction and self-perceptions of aging. Content and face validity were evaluated through expert panels and focus groups with participants of different ages. Exploratory factor analysis (EFA) was performed to analyze the dimensionality of the scale, reliability was assessed using internal consistency indices. Multi-Group Confirmatory Factor Analysis (MG-CFA) was used to test measurement invariance across gender and age groups.

**Results:**

The Spanish PAQ demonstrated a robust bidimensional structure, distinguishing between perceived negative and positive ageism, consistent with the original Dutch, Portuguese and Turkish versions. The internal consistency was acceptable for both subscales. MG-CFA confirmed full scalar invariance across gender and partial scalar invariance across age groups. Hierarchical regressions revealed that higher levels of perceived negative ageism were significantly associated with greater psychological distress (depression, anxiety and stress), lower life satisfaction and more negative self-perceptions of aging. Conversely, perceived positive ageism correlated with higher life satisfaction. Regarding age distribution, MIMIC models revealed a significant U-shaped trajectory for perceived negative ageism and an inverted U-shaped trajectory for perceived positive ageism across the lifespan.

**Conclusion:**

The Spanish version of the PAQ exhibits good psychometric properties, supporting its preliminary validity and reliability for assessing perceived age-based discrimination in Spanish-speaking populations across various age groups.

## Introduction

Ageism is a way of thinking, feeling and acting toward others or ourselves based on age, which involves stereotypes, prejudice and discrimination linked to how old someone is (World Health Organization, 2025). This type of discrimination is frequently perceived by people of different ages. For example, Chasteen et al. (2021) conducted a qualitative study with 208 participants from the United States, including young adults (18–35 years), middle-aged adults (36–59 years) and older adults (60+ years), who provided written descriptions of ageism experiences. All age groups reported frequent everyday ageism, particularly in workplaces; young adults noted it most often from coworkers doubting their competence, while middle-aged and older adults faced it more broadly when accessing goods/services, with young adults experiencing the highest relative frequency of such incidents. Likewise, Fowler and Gasiorek (2024) surveyed 231 young adults (18–29 years) in the United Kingdom via an online questionnaire, they found that over 75% of participants reported experiencing at least one instance of ageism during the preceding week, with more than 25% facing it weekly on average. Most instances of discrimination were regarding disrespect/being patronized or assumptions from middle-aged individuals in workplaces about their cognitive or social characteristics. Additionally, Allen et al. (2022a) analyzed a nationally representative U.S. sample of 2,035 adults aged 50–80 years old through an online survey, they found that 93.4% reported experiencing at least one form of everyday ageism regularly, with higher rates among older subgroups (e.g., more physical/mental health-related ageism in those 65+), though prevalence was similarly high across the 50–80 age range, showing no sharp drop-off by age within this group. Along the same lines, it has been found that perceived ageism appears similarly in women and men, despite possible differences in specific aspects (Ayalon and Bramajo, 2023; Allen, 2025).

Ageism has predominantly been conceptualized as a negative phenomenon (Chang et al., 2020), existing research has documented experiences in which older individuals perceive a societal devaluation of their contributions, often feeling that their activities are no longer viewed as valuable due to their age (Allen et al., 2022b). Furthermore, this negative impact extends to interactions motivated by ostensibly positive intentions. For instance, practices such as speaking more slowly to ensure a listener can follow the conversation are often categorized as benevolent or paternalistic ageism, as they reinforce age stereotypes in a more accepted way (Gans et al., 2023). Perceived ageism, characterized as the personal, everyday encounter of individuals with stereotypes, biases and discriminatory acts directed toward them because of their age (Brinkhof et al., 2022) is associated with poorer mental health. Different proposals have been identified to explain how ageism affects individual health. One of the most prominent is Levy's Stereotype Embodiment Theory (Levy, 2003, 2009), which posits that age stereotypes are learned from the surrounding culture since early in life (for example, perceiving ageism toward others or oneself) and, over time, become internalized, ultimately emerging as self-stereotypes. These beliefs can subsequently shape how individuals think, feel, and behave as they age—often without conscious awareness—influencing outcomes such as memory, depressive symptoms, physical functioning, and even longevity (Tully-Wilson et al., 2021). The theory further emphasizes that these effects become self-relevant in later life, as individuals begin to identify with the group targeted by age stereotypes. However, to the extent that stereotypes toward other age groups exist (for example, the belief that young people are lazy—a negative stereotype— or healthy —a positive stereotype—; World Health Organization, 2021), and to the extent that individuals have internalized them while considering themselves part of that age group, these stereotypes could also be associated with various health outcomes. In this regard, although most of the evidence on the association between perceived ageism and mental health has been examined in older adults, the literature is beginning to find evidence in younger people as well. For instance, in older adults, higher levels of perceived ageism were associated with greater symptoms of depression (Ayalon, 2018; Hajek and König, 2024; Kim et al., 2019; Sugisawa et al., 2025), anxiety (Shen et al., 2025) and stress (Patel et al., 2025), as well as lower life satisfaction (Hajek and König, 2024). Likewise, in middel-age adults, higher levels of perceived ageism were associated with greater symptoms of depression (Ayalon, 2018; Hajek and König, 2024; Sugisawa et al., 2025), and lower life satisfaction (Hajek and König, 2024). Finally, in young adults, higher levels of perceived ageism were associated with greater symptoms of depression (Sugisawa et al., 2025).

Despite this, the existence of positive perceived ageism linked to improved wellbeing has also been identified in recent years. Specifically, according to Brinkhof et al. (2022), perceived ageism is a multidimensional construct that distinguishes between negative and positive subjective experiences. Perceived negative ageism refers to persons' experiences of hostile or demeaning attitudes, such as being infantilized, ignored or unjustly treated as physically or mentally less capable due to their age. In contrast, perceived positive ageism involves being viewed through favorable stereotypes, where individuals are perceived as wise and sensible, their advice is valued for their life experience, and they are seen as meaningful contributors to society. The authors emphasize that recognizing this distinction is critical because, while negative ageism serves as a significant risk factor for physical and mental health, positive ageism can have beneficial impacts on mental wellbeing and cognitive functioning.

Following this multidimensional conceptualization of perceived ageism (positive and negative), Brinkhof et al. (2022) developed the Perceived Ageism Questionnaire (PAQ). The PAQ assesses perceived ageism, including stereotype, prejudice and discrimination dimensions, through two subscales measuring positive and negative forms of ageism. Ludwig et al. (2025) recently reviewed the available measures of perceived ageism, concluding that only the PAQ and the Nordic Age Discrimination Scale show acceptable psychometric support. While the Nordic Age Discrimination Scale (Furunes and Mykletun, 2010) focuses on analyzing ageism in the specific context of the workplace, the PAQ assesses the experience of perceived ageism in general, allowing for an exploration of the experience of perceived ageism outside the workplace as well.

Specifically, the PAQ (Brinkhof et al., 2022) assesses perceived ageism, including stereotype, prejudice and discrimination dimensions, through two subscales measuring positive and negative forms of ageism. This scale was first analyzed in Dutch (Brinkhof et al., 2022) in a sample of participants aged 55 to 93 years old, revealing a two-dimensional structure with good fit to the data and acceptable internal consistency. Subsequently, the psychometric properties of the Portuguese version of the PAQ (Miguel and Pedroso-Chaparro, 2025), with a sample of adults between 18 and 91 years of age, were analyzed, also showing a two-dimensional structure with good fit to the data and acceptable internal consistency. Recently, the Turkish adaptation of the scale (PAQ-TR), among older adults, aged 65 years and older, was analyzed by (Doğan and Doğan 2025), confirming a two-dimensional structure with an excellent fit to the data. The instrument showed generally acceptable internal consistency, although the positive subscale showed more limited reliability due to its short length. Additionally, the PAQ-TR showed excellent temporal stability with a test–retest Intraclass Correlation Coefficient of 0.91. The same bifactor structure of the PAQ, consisting of Factor 1 (perceived negative ageism) and Factor 2 (perceived positive ageism), was replicated across the Dutch, Portuguese, and Turkish versions. Consequently, all available psychometric evaluations of the PAQ across distinct linguistic and cultural groups have consistently converged on the same stable two-dimensional factor structure. Table 1 shows a summary of the psychometric properties demonstrated by the PAQ in the available versions.

**Table 1:** Perceived Ageism Questionnaire (PAQ) psychometric properties.

Version/References	Analysis	Factor 1 items	Factor 2 items	TLI	RMSEA [90% CI]	SRMR	α Negative ageism factor	α Positive ageism factor	α Perceived ageism (full scale)
**Dutch** (Brinkhof et al., 2022)	EFA	1, 2, 4, 6, 7	3, 5, 8	0.97	0.05 [0.03, 0.08]	0.03	0.79	0.71	0.67
	CFA			0.97	0.05 [0.03, 0.08]	0.02	0.81	0.75	0.63
**Portuguese** (Miguel and Pedroso-Chaparro, 2025)	EFA	1, 2, 4, 6, 7	3, 5, 8	0.96	0.06 [0.04, 0.07]	0.02	0.88	0.69	–
	CFA			0.96	0.06 [0.05, 0.07]	0.04	0.89	0.74	–
**Turkish** (Doğan and Doğan, 2025)	CFA	1, 2, 4, 6, 7	3, 5, 8	0.94	0.05 [0.01, 0.08]	0.03	0.72	0.51	–

EFA, exploratory factor analysis; CFA, confirmatory factor analysis; TLI, Tucker-Lewis Index; RMSEA, root mean square error of approximation; CI, confidence interval; SRMR, standardized root mean square residual; α = Cronbach's alpha.

Across the Dutch (Brinkhof et al., 2022), Portuguese (Miguel and Pedroso-Chaparro, 2025) and Turkish validation studies Doğan and Doğan, (2025), perceived negative ageism consistently correlates with higher psychological distress (including depression, anxiety and stress) and lower life satisfaction or quality of life. Moreover, findings from the Dutch and Turkish samples found that perceived negative ageism is associated with negative perceptions of aging, such as stronger negative consequences, heightened emotional representations and a more chronic awareness of the aging timeline. However, differences were found in the associations between perceived positive ageism and the variables assessed in the different versions. While all three versions link perceived positive ageism to higher life satisfaction, a key discrepancy emerges in the Portuguese study, where positive ageism was associated exclusively with life satisfaction and not with reduced distress. Furthermore, the Portuguese version uniquely identifies a U-shaped distribution of negative ageism across the lifespan, noting that younger and older adults perceive higher levels than those in middle age.

The PAQ has demonstrated robust psychometric properties across Dutch, Portuguese and Turkish versions, spanning diverse age groups. Furthermore, its associations with mental health indicators highlight its utility in understanding the impact of both negative and positive perceived ageism. Despite its international relevance, no validated Spanish version of this scale currently exists. Consequently, the primary objective of this study was to adapt the Perceived Ageism Questionnaire (PAQ) into Spanish and evaluate its psychometric properties. Additionally, this study aimed to examine potential age group or gender differences in perceived ageism.

## Method

### Participants

To analyze content validity, two focus groups composed by 11 adults who (1) were living in Spain and (2) were 18 years or older were created. After this first phase, 404 adults who (1) were living in Spain and (2) were 18 years or older completed a protocol that assessed the Spanish version of the PAQ along with other variables.

### Procedure

First, the English version of the Perceived Ageism Questionnaire (Brinkhof et al., 2022) was adapted to Spanish. The adaptation was carried out according to the guidelines of the International Test Commission (ITC, 2017; Muñiz et al., 2013). Regarding the translation process, two psychologists fluent in English and Spanish performed an independent and blinded translation of the original scale. Both translations were compared to the original (English) and Spanish versions of the PAQ. As a result, a consensus version of the instruments was developed.

This later version was assessed by two psychologists who were specialists in psychological assessment to determine content validity. They had to respond in terms of clarity (the item is easily understood, i.e., its syntax and semantics are adequate), relevance (the item is essential or important, i.e., it must be included) and that the changes respect the original version (justified/doubt/not justified). Additionally, a pilot study was conducted, in May 2024, to obtain evidence of face validity for expression clarity and content adequacy from the target population. Specifically, content validity evidence was obtained through two focus groups conducted with adults from different age cohorts. The first group included older adults (*n* = 6; ages 60–69; three women) and the second comprised middle-aged adults (*n* = 5; ages 31–39; three women). Participants evaluated the clarity and adequacy of each item of the Spanish adaptation of the PAQ using a 7-point scale.

Subsequently, the definitive version of the PAQ was distributed to a broader cohort of respondents to evaluate its psychometric characteristics. Participants were invited to join the study between April 2024 and May 2026 via an online survey hosted on the Qualtrics platform, which was shared through social media networks such as LinkedIn and Instagram. Additionally, paper-based questionnaires were self-administered at Madrid City Council centers offering cultural programs (e.g., gymnastics or painting) for seniors, specifically to bolster the representation of the older population within the sample.

Every participant finished the assessment and gave their informed consent, which detailed the voluntary nature of their involvement, guaranteed anonymity, ensured the absence of potential risks and provided details regarding the dissemination of results. This research received formal approval from the Ethics Committee of the Universidad a Distancia de Madrid.

### Measures

In addition to socio-demographic variables (sex, age, marital status, level of education and employment status), the following variables were assessed.

*Perceived Ageism* was measured using the Spanish translation of the Perceived Ageism Questionnaire (PAQ; Brinkhof et al., 2022), consisting of two subscales: perceived negative ageism and perceived positive ageism. The perceived negative ageism subscale is comprised of five items (e.g., “How often in the past year have you had the feeling that people hold negative prejudices or exaggerated stereotypes (e.g. weak, vulnerable, dull, slow) about you because of your age?”) and the perceived positive ageism subscale includes three items (e.g., “How often in the past year have you had the feeling that people see you as a meaningful part of society precisely because of your age?”). Both subscales are answered through a 5-point Likert scale ranging from 1 (“never”) to 5 (“very often”). The 8-item scale was translated into Spanish using the back-translation method. The psychometric properties of this scale, including factor analysis and Cronbach's alpha, are described in the Results section.

Self-perceptions of aging were measured by the Attitudes Toward One's Own Aging Subscale (Liang and Bollen, 1983), using the correction of Levy et al. (2002). It consists of five items (e.g., “As you get older, you are less useful”). Higher scores indicate higher levels of negative self-perceptions of aging. Cronbach's α in the present study was 0.64.

Depression, anxiety and stress symptoms were measured through subscales of the Spanish version (Ruiz et al., 2017) of the Depression Anxiety Stress Scale-21 (DASS-21; Lovibond and Lovibond, 1995). This scale is composed of three subscales with seven items each: depression (e.g., “I was unable to become enthusiastic about anything”), anxiety (e.g., “I found it difficult to relax”) and stress (e.g., “I was intolerant of anything that kept me from getting on with what I was doing”). The response format consists of a 4-point Likert scale ranging from 0 (“Did not apply to me at all”) to 3 (“Applied to me very much or most of the time”). Cronbach's α in the present study was 0.89, 0.86, and 0.87, for depression, anxiety and stress, respectively.

*Satisfaction with life* was measured using the Spanish version (Vázquez et al., 2013) of the Satisfaction With Life Scale (SWLS; Diener et al., 1985). The scale has 5-items (e.g., “The conditions of my life are excellent”). Response options consisted of a 5-point Likert scale, from 1 (“strongly disagree”) to 5 (“strongly agree”). Cronbach's α in the present study was 0.87.

### Data analysis

First, in the pilot study sample, Flies Kappa coefficient was performed to analyze content validity. To analyze the results of the pilot group about clearness and face validity of the PAQ, Aiken's V was calculated. In both cases, the results were interpreted based on percent agreement too.

Then, *a priori* power analysis was conducted using G^*^Power (v. 3.1.9.7) to determine the minimum sample size required for the study's statistical framework (Kang, 2021). The analysis was based on a Chi-square (*X*^2^) goodness-of-fit test for exploratory factor analysis, aiming for a medium effect size (Cohen's *w* = 0.30), an alpha level of α = 0.05 and a high statistical power (1–β = 0.95). The degrees of freedom were calculated through the standard EFA formula:


$$\begin{array}{l} df=\frac{1}{2}\cdot \left[{\left(p-m\right)}^{2}-\left(p+m\right)\right] \end{array}$$

where *p* was the number of items (*p* = 8) and *m* represents the number of extracted factors *m* = 2; expected based on the original structure by Brinkhof et al. (2022), resulting in df = 13. The analysis indicated a minimum required sample size of *N* = 295. This sample size ensures a 95% probability of detecting the hypothesized effect, resulting in an actual power of 0.9500 and a critical *X*^2^ value of 22.36.

Among participants who completed the final version of the PAQ, descriptive statistics including means, standard deviations and ranges were computed for both sample and item characteristics. Subsequently, the dimensionality of the Spanish adaptation of the PAQ was investigated. Suitability of the sample data for factor analysis was first evaluated through KMO and Bartlett's test of sphericity. Multiple approaches were applied to determine the number of factors needed to account for the observed correlations. Parallel analysis was conducted (Revelle, 2017), specifically applying the mean and 95th percentile eigenvalue criteria proposed by Longman et al. (1989). A Geomin oblique rotation was implemented. The Maximum Likelihood Robust (MLR) estimator was utilized, yielding parameter estimates identical to those from Maximum Likelihood (ML) alongside robust standard errors and a scaled chi-square statistic that is more resilient to non-normality (Muthén and Muthén, 2017). Model fit was evaluated using several indices: the chi-square test (χ^2^) and standardized root mean square residual (SRMR) as absolute measures; comparative fit index (CFI) and Tucker-Lewis index (TLI) as incremental indices; and root mean square error of approximation (RMSEA) with its 90% confidence interval as a parsimony index. Per Hu and Bentler (1998), values of CFI and TLI near or higher than 0.95, SRMR near or lower than 0.08, and RMSEA near or lower than 0.06 indicate acceptable model fit. Finally, the magnitude of the factor loadings of 0.30 was the cutoff value used to determine each item contribution to their respective factor (Cudeck and O'Dell, 1994). Reliability was examined via internal consistency using Cronbach's alpha (α) and McDonald's omega (ω) coefficients. Following established psychometric guidelines, values ranging from 0.70 to 0.95 are considered indicative of good internal consistency (Tavakol and Dennick, 2011). However, for shorter subscales coefficients starting from 0.60 are considered moderate and acceptable (Streiner, 2003). To account for statistical uncertainty, 95% confidence intervals were calculated and reported for all reliability estimates (Oosterwijk et al., 2019).

Then, in order to determine if the scale exhibits measurement invariance across the different age groups —young adults (18–39 years old), middle-aged adults (40–64 years old), and older adults (65 and older)— as well as between women and men, a Multi-group Confirmatory Factor Analysis was conducted following the sequential levels described by Putnick and Bornstein (2016): configural invariance (equivalence of model form), metric invariance (equivalence of factor loadings), and scalar invariance (equivalence of item intercepts or thresholds). To evaluate the fit between these nested models, we followed the guidelines of Xu and Tracey (2017), who state that simulation studies comparing multiple goodness-of-fit indices (e.g., chi-square, AIC, RMSEA, and CFI) have recommended ΔCFI as it is independent of model complexity and sample size, and a ΔCFI < 0.01 indicates invariance. In instances where full measurement invariance was not supported, the pursuit of partial invariance was conducted to ensure valid cross-group comparisons. To identify specific items responsible for the lack of equivalence, we utilized the backward modification index method. Following the scientific recommendations of Jung and Yoon (2016), a conservative statistical cut-off value of 6.635 was applied to the Modification Indices (M.I.) to identify parameters that significantly degraded model fit. The adjustment of the models followed the logic introduced by Sörbom (1989), where parameters with the highest M.I. values—reflecting the greatest source of statistical tension in the model—were considered for sequential liberation. However, in accordance with the standards of Putnick and Bornstein (2016) and Sörbom (1989), statistical indices were not followed blindly. Instead, the final decision to release a constraint was based on a combination of the highest M.I. value and a substantive review by experts, who evaluated the item content to ensure that any detected non-invariance was theoretically meaningful and interpretably sound in the context of the ageism construct.

In order to analyse age distribution of perceived negative and positive ageism, following the establishment of partial scalar invariance between different age groups (achieved by freeing the intercept of Item one, eight and two to ensure that latent mean comparisons were not biased by measurement artifacts), a latent regression approach (MIMIC modeling) was implemented to evaluate the presence of perceived negative and positive ageism across different ages. These analyses were utilized instead of discrete group comparisons (such as Kruskal–Wallis or Mann–Whitney *U*-tests) because treating age as a continuous predictor allows for the statistical confirmation of non-linear relationships.

Then, in order to analyse gender distribution of perceived negative and positive ageism, given that the Kolmogorov–Smirnov *Z*-tests indicated that the data for all criteria significantly deviated from normality (all *p* < 0.01), the Mann–Whitney *U*-test (Chicco et al., 2025) was employed for comparisons involving women and men.

Finally, to evaluate the convergent validity of the Perceived Ageism Questionnaire (PAQ), a series of two-step hierarchical regression analyses were conducted. These analyses examined the predictive power of perceived ageism on assessed variables: negative self-perceptions of aging, symptoms of depression, anxiety, and stress, as well as satisfaction with life. Given that the Kolmogorov–Smirnov *Z*-tests indicated that the data for all criteria significantly deviated from normality (all *p* < 0.01), a bootstrapping procedure with 1,000 resamples was employed to obtain robust standardized coefficients (β) and 90% confidence interval. This approach is particularly suitable when full measurement invariance cannot be established and small group-specific sample sizes—such as those observed in the older adult group—limit the feasibility of more complex multigroup structural models.

Analyses were performed with IBM SPSS Statistics (v.22.0) and Mplus 7.0 (Muthén and Muthén, 2012).

## Results

### Content and face validity

A panel of two experts reviewed the PAQ to determine whether items were clear and relevant and to justify changes concerning its theoretical background. There was an agreement rate of 100% between the experts for the clarity and relevance dimensions, with a value of Fleiss Kappa of one.

Discussion groups with older and younger people to assess face validity and to clarify the items shows, for the older adult group, an excellent Aiken V coefficient (*V* = 1) for all items. For the younger group, the results showed very good Aiken V coefficients (*V* = 1) in all items except for items one (V = 0.97), 3 (V = 0.87), and 6 (V = 0.90). Following this procedure, minor adjustments were made to the phrasing and word order of specific items within the questionnaire. In terms of face validity, the pilot study revealed no concerns regarding clarity or acceptability among participants; consequently, no additional modifications were required. The final set of items is presented in Table 2.

**Table 2:** Factor loadings, means, standard deviations and ranges of the Perceived Ageism Questionnaire questions.

How often in the past year have you had the feeling that...	EFA Component [90% CI] Factor 1	EFA Component [90% CI] Factor 2
1. ... people approach you as if you are a child because of your age?	0.531 [0.434, 0.628]	−0.110 [−0.236, 0.016]
(1.) *... la gente se comporta contigo como si fueses un niño/a (es decir, de un modo infantil) debido a tu edad?*		
2. ... you are not being listened to and/or your opinion/advice is not taken seriously because of your age?	0.662 [0.577, 0.748]	−0.121 [−0.232, −0.011]
(2.) *... no eres escuchado/a y/o tu opinión no se tiene en cuenta debido a tu edad?*		
3. …people value your advice and contribution to a conversation because of our life experience?	−0.082 [−0.198, 0.035]	0.616 [0.512, 0.720]
(3.) *... la gente valora tu consejo y aportaciones a una conversación debido a tu experiencia vital?*		
4. ... people hold negative prejudices or exaggerated stereotypes (e.g., weak, vulnerable, dull, slow) about you because of your age?	0.651 [0.561, 0.742]	0.018 [−0.067, 0.102]
(4.) *... la gente tiene prejuicios negativos (p. ej., débil, vulnerable, aburrido, lento) sobre ti debido a tu edad?*		
5. ... people assume that you are wise and sensible because of your age?	0.000 [−0.005, 0.004]	0.669 [0.578, 0.759]
(5.) *... la gente asume que eres sabio/a debido a tu edad?*		
6. ... people unjustly treat you as if you are less capable (mentally or physically) because of your age?	0.730 [0.653, 0.807]	0.008 [−0.053, 0.069]
(6.) *... la gente te trata como si fueras menos capaz (mental o físicamente) debido a tu edad?*		
7. ... people think disparagingly (with little or no dignity) about your contribution to society because of your age?	0.648 [0.577, 0.720]	−0.001 [−0.040, 0.038]
(7.) *... la gente valora de manera despectiva tus aportaciones actuales a la sociedad debido a tu edad?*		
8. ... people see you as a meaningful part of society precisely because of your age (e.g., babysitting (grand) children or volunteer activities)?	0.076 [−0.018, 0.170]	0.621 [0.526, 0.716]
(8.) *... la gente te ve como una parte relevante de la sociedad, precisamente debido a tu edad?*		
Mean	8.67	9.30
S.D.	3.19	2.37
Range	5–20	3–15
Model fit	*X*^2^ (28) = 511.90, *p* = 0.000; RMSEA = 0.05 CI 90% [0.025–0.081]; CFI = 0.969; TLI = 0.934; SRMR = 0.028	

Factor 1: perceived negative ageism; Factor 2: perceived positive ageism.

### Sample characteristics

Participants were aged between 19 and 95 (mean age = 47.93; SD = 17.52) and were mainly women, married or with a partner, with a bachelor's degree and employed, as Table 3 shows.

**Table 3:** Sample characteristics.

Sample characteristics	Total sample (*n* = 404)	Age group			Gender group	
		Young adults: 18–39 years (*n* =146)	Middle-aged adults: 40–64 years (*n* = 171)	Older adults: 65+ years (*n* =87)	Female (*n* = 308)	Male (*n* = 92)
Age, M (SD) [Range]	47.93 (17.52) [18–95]	29.42 (6.55) [18–39]	51.11 (7.11) [40–64]	72.72 (6.50) [65–95]	47.29 (17.43) [18–95]	49.98 (17.42) [18–82]
Gender						
Female	76.2%	78.8%	77.8%	69%	100%	0%
Male	22.8%	29.9%	22.2%	28.7%	0%	100%
Other/Not reported	1%	1.4%	0%	2.3%	0%	0%
Marital status						
Single	33.2%	66.4%	19.3%	4.6%	33.4%	32.6%
Married/partner	50.2%	31.5%	66.1%	50.6%	48.4%	57.6%
Divorced/separated	9.7%	1.4%	12.9%	17.2%	10.7%	6.5%
Widow	6.9%	0.7%	1.8%	27.6%	7.5%	3.3%
Level of education						
Basic education	4.4%	0.7%	2.3%	14.9%	4.5%	4.3%
Secondary education	5.4%	0.7%	4.1%	16.1%	5.5%	5.4%
Bachelor's degree	29.2%	26%	30.4%	32.2%	28.6%	31.5%
University degree	29.5%	32.9%	28.1%	26.4%	29.9%	28.3%
Master's degree	24.3%	34.2%	25.7%	4.6%	24.7%	23.9%
Doctorate	5.2%	3.4%	8.8%	1.1%	5.2%	5.4%
Other/not reported	1.9%	2.1%	0.6%	4.5%	1.6%	1.1%
Employment status						
Employed	41.8%	41.1%	62.6%	2.3%	42.9%	39.1%
Unemployed	8.2%	8.2%	12.3%	0%	8.8%	6.5%
Student	11.1%	26%	4.1%	0%	12%	8.7%
Student worker	11.1%	19.9%	9.4%	0%	11.4%	9.8%
Retired	22.5%	0%	4.7%	95.4%	20.5%	28.3%
Other/not reported	5.2%	4.8%	7.0%	2.3%	4.5%	7.6%
Face-to-face questionnaire	11.6%	0.5%	1.2%	50.6%	11.7%	10.9%

### Exploratory factor analysis

**Figure 1:**
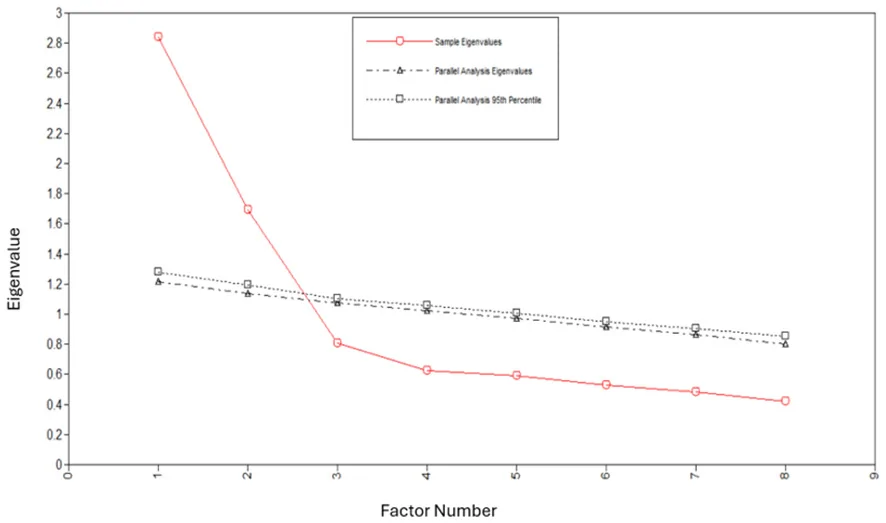
Parallel analyses using polychoric correlation matrix.

The KMO (0.768) and Bartlett sphericity test (*X*^2^ = 750.65, *p* < 0.001) showed the data to be highly suited for factor analysis. Parallel analyses suggested a two-factor solution was the optimal number of factors for the PAQ. The two empirical eigenvalues (2.843 and 1.695, calculated from principal component analysis) were larger than the simulated eigenvalues (See Figure 1). Consequently, two factors were retained. The first factor was labeled as “Perceived Negative Ageism,” and the second one was labeled as “Perceived Positive Ageism,” including the same items in each factor as the original version (Brinkhof et al., 2022). The bidimensional model explained 56.73% of the variance of the assessed construct and had an excellent fit to the data: *X*^2^ (28) = 511.90, *p* = 0.000; RMSEA = 0.05 CI 90% [0.025–0.081]; CFI = 0.969; TLI = 0.934; SRMR = 0.028. All factor loadings were equal to or higher than 0.50 (Table 2), showing that all items were reliably observed indicators of the measured dimension.

### Reliability

The internal consistency of the Spanish version of the PAQ scale was measured through Cronbach's α and was 0.72 CI 95% [0.67–0.76], while the Cronbach alpha values of the Perceived negative ageism and Perceived positive ageism domains were 0.79 CI 95% [0.76–0.82] and 0.66 CI 95% [0.60–0.71], respectively. The McDonald's ω coefficient for the overall scale was 0.69 CI 95% [0.63–0.73] and those for the Perceived negative ageism and Perceived positive ageism domains were 0.79 CI 95% [0.75–0.83] and 0.67 CI 95% [0.60–0.73], respectively.

### Measurement invariance across age groups

Following a sequential constraint approach, a series of nested models within a bifactor framework was estimated. Initially, a configural invariance model (Model 1) was tested to evaluate whether the factor structure was equivalent across groups without imposing equality constraints on parameters. As shown in Table 4, Model 1 displayed an adequate fit to the data (CFI = 0.939; RMSEA = 0.069), suggesting that the bifactor structure of the PAQ is consistent across young, middle-aged, and older adults. Next, metric invariance (Model 2) was examined by constraining factor loadings to be equal across groups. Although the model fit remained acceptable (CFI = 0.905; RMSEA = 0.078), the decrease in CFI compared to the configural model exceeded the recommended threshold (ΔCFI = 0.034 > 0.010), suggesting some degree of non-invariance in the factor loadings. However, following the sequential procedure for identifying scalar equivalence, Model 2 was used as the baseline for testing the more restrictive scalar models. Regarding full scalar invariance (Model 3), the model was strictly rejected due to poor fit (CFI = 0.804; RMSEA = 0.104) and a substantial drop in adjustment (ΔCFI = 0.101). To identify the sources of this misfit, Modification Indices (M.I.) were examined. The results for Model 3 pointed to Item 1 (M.I. = 30.429 in the young group) and Item 8 (M.I. = 20.076 in the young group and 12.769 in the middle-aged group) as the primary sources of non-invariance. Consequently, partial scalar invariance was pursued following a sequential “one-by-one” liberation protocol. Initially, the intercept of Item 1 was freed (Model 3a), but the adjustment remained insufficient (CFI = 0.856), with the M.I. still highlighting Item 8 (M.I. = 20.070) as a major source of bias. In the subsequent step, freeing the intercept of Item 8 (Model 3b) significantly improved the model (CFI = 0.891), yet a significant M.I. remained for the intercept of Item 2 (M.I. = 12.415 in the young group). Finally, after freeing the intercept of Item 2 (Model 3c), the model achieved a satisfactory fit (CFI = 0.908; RMSEA = 0.074) and the change in CFI relative to the metric model was eliminated (ΔCFI = −0.003), fulfilling the criteria for partial scalar invariance. At this stage, no other intercept modification indices exceeded the critical threshold.

**Table 4:** Goodness-of-fit indices for the different invariance bifactor models across age groups.

Model	Number of parameters	RMSEA [90% CI]	AIC	CFI	Δ CFI	TLI
Model 1: configural metric	75	0.069 [0.043–0.094]	7,776.309	0.939	–	0.910
Model 2: metric invariance	63	0.078 [0.056–0.100]	7,793.516	0.905	0.034	0.884
Model 3: scalar Invariance	51	0.104 [0.086–0.122]	7,847.267	0.804	0.101	0.796
Model 3a: partial scalar invariance [*ν1*]	53	0.090 [0.071–0.109]	7,814.736	0.856	0.049	0.847
Model 3b: partial scalar invariance [*ν1, ν8*]	55	0.079 [0.059–0.100]	7,794.686	0.891	0.014	0.881
Model 3c: partial scalar invariance [*ν1, ν8, ν2*]	57	0.074 [0.052–0.095]	7,784.822	0.908	−0.003	0.897

ν*1* intercept of Item 1, ν*8* intercept of Item 8, ν*2* intercept of Item 2.

### Measurement invariance across gender groups

Initially, a configural invariance model (Model 1) was tested to evaluate whether the factor structure was equivalent across gender groups without imposing equality constraints on parameters. As shown in Table 5, Model 1 displayed an excellent fit to the data (CFI = 0.979; RMSEA = 0.069), indicating that the bifactor structure of the PAQ is equivalent for both men and women. Next, metric invariance (Model 2) was examined by constraining factor loadings to be equal across groups. The model maintained a good overall fit (CFI = 0.965; RMSEA = 0.069), although the decrease in CFI relative to the configural model was slightly above the conventional threshold (ΔCFI = 0.014 > 0.010). Despite this marginal drop, the global fit indices remained well within acceptable limits, and Model 2 was retained as the baseline for testing the more restrictive scalar model. Regarding full scalar invariance (Model 3), the model showed a highly satisfactory fit (CFI = 0.963; RMSEA = 0.050). Most importantly, the change in CFI relative to the metric model was negligible (ΔCFI = 0.002), fulfilling the strict criterion for invariance (ΔCFI < 0.010). Then, item intercepts are equivalent across men and women, suggesting that differences in item means are due to differences in latent factor levels rather than measurement bias.

**Table 5:** Goodness-of-fit indices for the different invariance bifactor models across gender.

Model	Number of parameters	RMSEA [90% CI]	AIC	CFI	Δ CFI	TLI
Model 1: configural metric	48	0.069 [0.043–0.094]	7,875.763	0.979	–	0.971
Model 2: full metric invariance	43	0.069 [0.043–0.094]	7,873.721	0.965	0.014	0.956
M3: full scalar invariance	46	0.050 [0.020–0.074]	7,877.835	0.963	0.002	0.950

### Differences between age groups in perceived ageism

**Figure 2:**
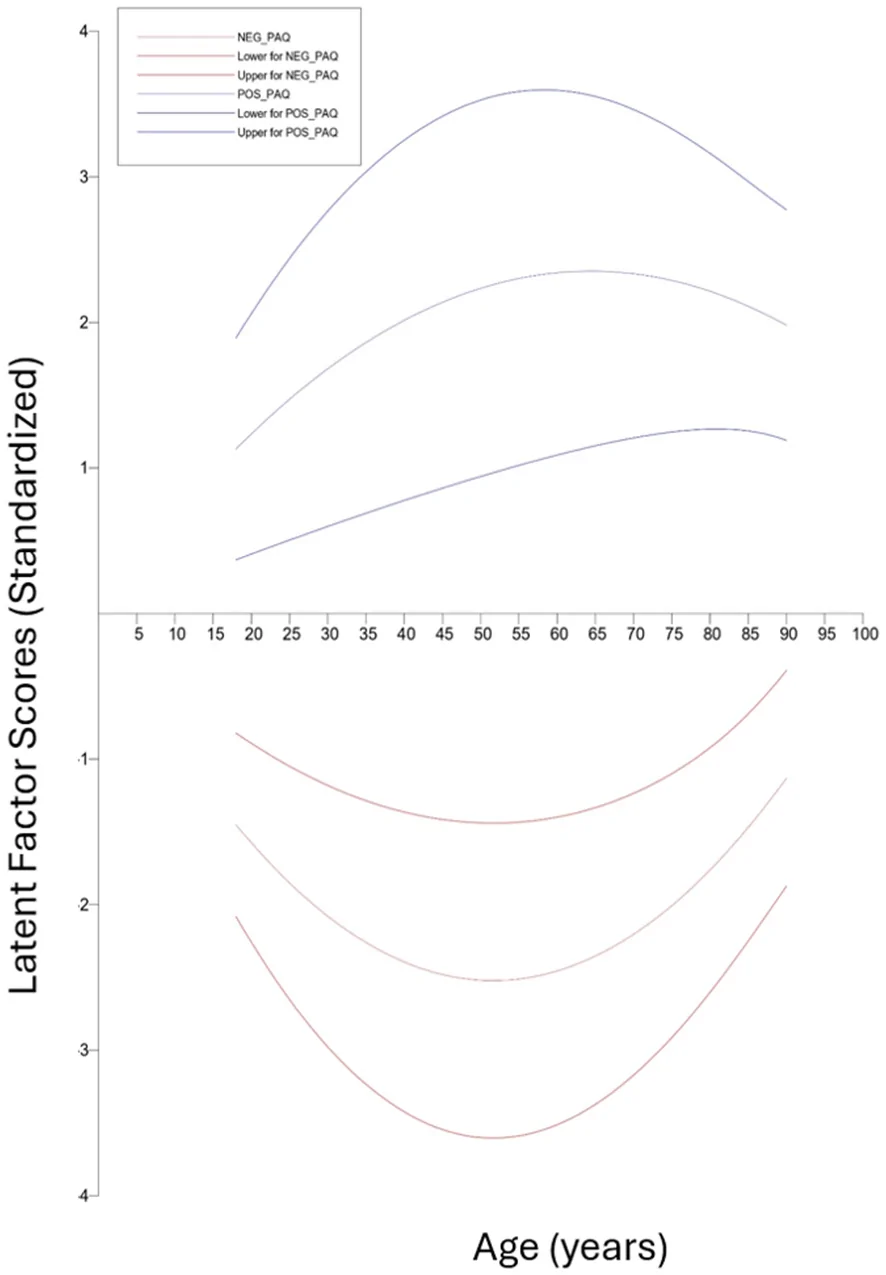
Latent trajectories of perceived ageism from age. The lines represent the predicted levels of Perceived Negative Ageism (NEG_PAQ) and Perceived Positive Ageism (POS_PAQ) derived from the MIMIC model. The graph illustrates a U-shaped distribution for negative ageism, with higher levels reported by the youngest and oldest participants, and an inverted U-shape for positive ageism, which peaks during middle adulthood. Predicted values are adjusted for differential item functioning in non-invariant indicators.

As shown in Table 6, to address potential non-linearity in the experience of ageism, a MIMIC model was estimated to evaluate the trajectories of perceived negative and positive ageism across the lifespan while controlling for item-level measurement bias (items 1, 2, and 8). The model yielded an adequate fit (*X*^2^ (28) = 61.18, *p* = 0.000; RMSEA = 0.06 CI 90% [0.037–0.073]; CFI = 0.941; TLI = 0.907; SRMR = 0.032) and confirmed significant quadratic effects for both factors. Specifically, Perceived Negative Ageism followed a significant U-shaped trajectory ($\beta_{age}^{2}$ = 0.001, *p* < 0.001), characterized by higher levels in youth and older age, with a distinct dip during midlife. Conversely, Perceived Positive Ageism exhibited an inverted U-shape ($\beta_{age}^{2}$ = −0.001, *p* = 0.037), increasing toward middle age before leveling off (see Figure 2).

**Table 6:** MIMIC model: linear and quadratic effects of age on latent ageism factors.

Latent factor	Predictor	Estimate	S.E.	*p*-value
Negative ageism (F1)	Age (linear)	−0.098	0.022	< 0.001
	Age^2^ (quadratic)	0.001	0.000	< 0.001
Positive ageism (F2)	Age (linear)	0.073	0.026	0.006
	Age^2^ (quadratic)	−0.001	0.000	0.037
Model fit	*X*^2^ (28) = 61.18, *p* = 0.000; RMSEA = 0.06 CI 90% [0.037–0.073]; CFI = 0.941; TLI = 0.907; SRMR = 0.032			

Estimates are unstandardized. Model controls for DIF in items 1, 2, and 8.

### Differences between gender groups in perceived ageism

No significant differences were found in levels of negative perceived ageism or positive perceived ageism between women and men, as shown in Table 7.

**Table 7:** Differences between women and men in negative perceived ageism and positive perceived ageism.

Variable	Measure	Total sample (*n* = 400)	Group 1 women (*n* = 308)	Group men (*n* = 92)	Mann–Whitney *U*-test
Perceived negative ageism	Median IQR	8	8	8	13,955.5 (*p* = 0.826)
		6–11	6–11	6–10	
Perceived positive ageism	Median IQR	9	9	9	14,074.5 (*p* = 0.923)
		8–11	8–11	8–11	

IQR, Interquartile range.

### Construct validity

Table 8 presents the results concerning the impact of perceived ageism on negative self-perceptions of aging, symptoms of depression, anxiety, and stress, as well as satisfaction with life. In Step 1, chronological age was entered as a control variable, accounting for a negligible amount of variance across most criteria (*R*^2^ ≤ 0.050) and exhibiting a significant association only with stress symptoms (β = −0.06, *p* < 0.01). In Step 2, the addition of the PAQ factors significantly improved model fit for all outcomes, with Δ*R*^2^ values ranging from 0.059 to 0.125 (all *p* < 0.01).

**Table 8:** Hierarchical regression models.

Variables	Negative self-perceptions of aging^a^		Depression symptoms		Anxiety symptoms		Stress symptoms		Satisfaction with life^a^	
	*Step 1 β* [CI 90%]	*Step 2 β* [CI 90%]	*Step 1 β* [CI 90%]	*Step 2 β* [CI 90%]	*Step 1 β* [CI 90%]	*Step 2 β* [CI 90%]	*Step 1 β* [CI 90%]	*Step 2 β* [CI 90%]	*Step 1 β* [CI 90%]	*Step 2 β* [CI 90%]
Age	0.00 [−0.01, −0.01]	0.01 [−0.01, 0.02]	−0.02^*^ [−0.04, −0.00]	−0.01 [−0.03, 0.01]	−0.03^*^ [−0.05, −0.01]	−0.02 [−0.04, 0.01]	−0.06^**^ [−0.08, −0.03]	−0.04^*^ [−0.06, −0.02]	0.02 [−0.02, 05]	−0.00 [−0.03, 0.03]
Perceived negative ageism		0.13^**^ [0.06, 0.19]		0.35^**^ [0.23, 0.47]		0.41^**^ [0.27, 0.55]		0.41^**^ [0.26, 0.57]		−0.36^**^ [−0.52, −0.19]
Perceived positive ageism		−0.03 [−0.12, 0.05]		−0.09 [−0.25, 0.06]		0.00 [−0.17, 17]		−0.04 [−0.22, 0.14]		0.49^**^ [0.25, 0.77]
*R* ^2^	0.000	0.059	0.010	0.096	0.013	0.114	0.046	0.126	0.005	0.130
Δ*R*^2^		0.059		0.085		0.101		0.080		0.125
*F*		*F*(3) = 17.23 (*p* < 0.01)		*F*(3) = 14.10 (*p* < 0.01)		*F*(3) = 17.15 (*p* < 0.01)		*F*(3) = 19.17 (*p* < 0.01)		*F*(3) = 14.66 (*p* < 0.01)
Kolmogorov–Smirnov Z	2.89 (*p* < 0.01)		3.83 (*p* < 0.01)		3.29 (*p* < 0.01)		1.70 (*p* < 0.01)		1.77 (*p* < 0.01)	
Mean	3.12		10.27		10.66		13.39		18.77	
SD	1.71		3.82		4.04		4.57		4.79	
Range	1–7		7–28		7–27		7–28		5–25	

^*^p < 0.05; ^**^p < 0.01.

Bootstrapping procedure with 1,000 resamples was employed.

^a^The analyses using the variables Negative self-perceptions of aging and Satisfaction with life were conducted with a sample size of 300 participants due to missing data for these variables in the assessment.

Regarding individual criteria, the inclusion of the PAQ factors explained an additional 5.9% of the variance in negative self-perceptions of aging (Δ*R*^2^ = 0.059, *p* < 0.01), with Perceived Negative Ageism acting as the sole significant predictor (β = 0.13, *p* < 0.01). For depressive symptoms, these factors contributed to an 8.5% increase in explained variance (Δ*R*^2^ = 0.085, *p* < 0.01), where Perceived Negative Ageism acted as a robust positive predictor (β = 0.35, *p* < 0.01). This factor was also a critical predictor of higher anxiety (β = 0.41, *p* < 0.01), with the model accounting for 11.4% of the total variance. Similarly, for stress symptoms, Perceived Negative Ageism emerged as a strong predictor (β = 0.41, *p* < 0.01), and the total variance explained by the model reached 12.6%. Finally, the satisfaction-with-life model showed the highest increment in explained variance (Δ*R*^2^ = 0.125, *p* < 0.01), with both factors emerging as significant predictors: Perceived Negative Ageism was negatively associated with satisfaction (β = −0.36, *p* < 0.01), while Perceived Positive Ageism was a strong and unique positive predictor (β = 0.49, *p* < 0.01).

## Discussion

The main objective of this study was to develop and analyze the Spanish version of the Perceived Ageism Questionnaire comprised of two subscales (Perceived Negative Ageism and Perceived Positive Ageism; Brinkhof et al., 2022). To this end, after conducting a back-translation process of the items, two experts analyzed the clarity and relevance of the items. Subsequently, two discussion groups were held so that participants of different ages could express their opinions on the clarity of the items and make proposals to modify them and agree on the changes with the group. Following this process, an exploratory factor analysis of the scale was performed, which suggested that the Spanish version of the PAQ shows good psychometric properties. Specifically, both the factor solution and the model fit and internal consistency presented by the scale were consistent with previous data shown by the Dutch (Brinkhof et al., 2022), Portuguese (Miguel and Pedroso-Chaparro, 2025) and Turkish (Doğan and Doğan, 2025) versions. It is important to note that, from a psychometric perspective, the observed reliability for the positive subscale (α = 0.66) in the Spanish PAQ —which is similar to that reported in studies of previous versions of the PAQ— is directly influenced by its brevity. However, in accordance with established methodological guidelines, for short subscales or those used in exploratory research, internal consistency coefficients of 0.60 or higher are considered moderate and acceptable (Streiner, 2003).

Additionally, in the Spanish version of the PAQ Multi-group CFA confirmed full scalar invariance across gender and partial scalar invariance across age groups by freeing the intercepts of items 1, 8, and 2. A potential explanation for the partial invariance detected in Items 1, 2, and 8 is that different age groups use distinct psychological reference frames to interpret these experiences. This differential interpretation according to social group membership, in this case age group, can be explained by Social Identity Theory (Tajfel, 1981), according to which individuals process social information in ways that protect their self-identity in relation to the group to which they belong. From this perspective, in Items 1 and 2, while younger adults may interpret being “treated like a child” or “not being listened to” as a sign of low status based on a stereotype of inexperience (for example, frequently experienced in the work context; Petery and Grosch, 2022), older adults may associate these experiences with assumptions of cognitive decline or with instances of elderspeak, a phenomenon frequently reported by older populations (Shaw and Gordon, 2021). In addition, a plausible explanation for the lack of invariance in Item 8 (“...people see you as a meaningful part of society precisely because of your age”) is the use of productivity as a frame of reference. This conceptualization suggests that the interpretation of “social relevance” is closely tied to labor market status and economic utility. Specifically, the idea that being a meaningful member of society is closely linked to formal employment and labor productivity is examined within the construct of productive aging. In industrial societies, employment functions as the main social anchor, so retirement or exclusion from the labor market often translates into a loss of identity and social relevance (Tamers et al., 2018). For example, it has been shown that older adults may struggle to remain in the labor market precisely in order not to lose their social value and autonomy, as discussed by Drazic et al. (2023).

With regards to the distribution of ageism levels according to age group, our results align with those previously found in the Portuguese sample (Miguel and Pedroso-Chaparro, 2025) regarding perceived negative ageism, confirming a significant U-shaped distribution. This trajectory is characterized by higher levels of reported perceived negative ageism in both younger and older participants, with a distinct dip during middle age. However, for perceived positive ageism, our results reveal a nuanced inverted U-shaped trajectory, where perceptions of positive ageism increase toward middle age before showing a slight stabilization or decline in the oldest groups. This differs from the linear upward trend suggested by the Portuguese validation. These differences in the distribution of positive and negative ageism between the data from the present study and the data from the study conducted by Miguel and Pedroso-Chaparro (2025) may be due to sampling strategies. While the participants evaluated by Miguel and Pedroso-Chaparro (2025) responded in all cases to an online survey, in the case of the present study, the difficulty in accessing older participants through social media led to an increase in the number of older participants through face-to-face sampling in community centers offering activities for older adults, given that few responses were obtained online. Studies suggest a consistent prevalence of ageism on social media (Dickinson et al., 2024), therefore, the sample in this study, which was less familiar with the use of social media, could be exposed to lower levels of ageism. This may particularly affect perceived positive ageism, as older adults who create content for these media often portray themselves through positive ageist stereotypes (they are ten times more likely to portray themselves using these stereotypes than negative ones; Ng and Indran, 2023). Moreover, consistent with previous studies (Ayalon and Bramajo, 2023; Allen, 2025), the results of the present study show no significant differences in perceived ageism reported between men and women. Despite this and considering that the use of this scale has previously found that men reported a greater perception of negative ageism (Miguel and Pedroso-Chaparro, 2025), it is possible that in the present study, the low participation of men was insufficient to make a robust comparison between the two groups.

Additionally, an association between higher perceived negative ageism and higher negative self-perception of aging was found through hierarchical regression models that controlled for chronological age and included both PAQ dimensions simultaneously. In contrast, no association was found between positive perceived ageism and negative self-perception of aging. These results are consistent with those found in previous studies using the Dutch version (Brinkhof et al., 2022), in which associations between positive perceived ageism and self-perception of aging only occured in certain dimensions, and the Turkish version (Doğan and Doğan, 2025), in which there was no significant association between positive perceived ageism and self-perception of aging versions of the scale. These results can be explained by considering both the stereotype embodiment model proposed by Levy (2003, 2009) and negativity bias (Norris, 2021). On the one hand, according to the framework established by Levy (2003, 2009), attitudes toward aging originate as external stereotypes that are reinforced during adulthood. It is essential to note that these biases end up being assimilated into the individual's identity, moving from general social observations to subjective stereotypes that define one's own experience of aging. For example, perceiving ageism in the workplace has been associated with a worse perception of aging (Takeuhi and Katagiri, 2024). On the other hand, according to negativity biases, we know that negative information has a stronger impact than positive information on processes such as attention, perception, memory and decision-making (Norris, 2021). For example, Salsano et al. (2024) found that negative emotions increase the accuracy and speed of memory-guided visual search, with stronger effects than positive/neutral emotions. Therefore, the results of this study could be explained by considering that people's perception of their own aging is influenced by the ageism they experience, with experiences of perceived negative ageism having a greater impact than experiences of perceived positive ageism.

Furthermore, the results were consistent with previous studies (Brinkhof et al., 2022; Doğan and Doğan, 2025; Miguel and Pedroso-Chaparro, 2025), showing that higher reported levels of perceived negative ageism were associated with higher levels of symptoms of depression, anxiety, stress and lower satisfaction with life. Notably, the use of a hierarchical framework revealed the unique predictive power of each dimension beyond chronological age, with the PAQ factors providing a significant incremental contribution to the explained variance across all criteria. Likewise, as previously shown in other studies, the associations between perceived positive ageism and emotional distress were less consistent in this sample. Specifically, the present study found no significant associations between perceived positive ageism and symptoms of depression, stress, or anxiety. On the contrary, as in previous studies (Brinkhof et al., 2022; Doğan and Doğan, 2025; Miguel and Pedroso-Chaparro, 2025), an association was found between higher reported levels of perceived positive ageism and greater satisfaction with life. These results may be consistent with greater attention to and greater recall of experiences of negative ageism, in line with the negativity bias (Norris, 2021), limiting the protective impact of experiences of positive ageism on emotional distress while still allowing for a positive impact on the cognitive evaluation of one's life.

Furthermore, the results of this study have several implications. First, our results suggest that perceived negative ageism and perceived positive ageism are two distinct dimensions rather than opposite poles of a single continuum. This distinction carries important theoretical and methodological implications, as it breaks with the classical assumption of mutual exclusivity. This independence implies that the absence of perceived negative ageism does not necessarily predict the presence of perceived positive ageism. In support of this differentiation, our hierarchical regression models provide robust evidence: by evaluating the unique predictive power of each dimension while controlling for chronological age and the other PAQ factor, we identified differentiated consequences for each construct. More specifically, while perceived negative ageism was a robust predictor of emotional distress and negative self-perceptions, perceived positive ageism emerged as a strong predictor of life satisfaction. These distinct patterns of association suggest that both dimensions possess unique underlying mechanisms and clinical implications. Therefore, this study reinforces the necessity for researchers and clinicians to evaluate both dimensions separately through independent scores rather than a global net score, a shift essential for designing tailored interventions and capturing the true complexity of the aging experience. Second, they show the importance of having measures of perceived ageism, both for older and younger populations, suggesting that it is the younger population that could be particularly affected by negative ageism. It also supports previous work that has already begun to advocate for the need to promote a less stereotypical view of both older and younger people, suggesting the need to promote practices in psychology, education and public policy (Wray-Lake et al., 2025).

Nevertheless, several limitations of the Spanish validation of the PAQ should be acknowledged. First, the use of a non-probabilistic convenience sampling strategy with a small sample size may limit the generalizability of the findings to the broader Spanish population. Furthermore, unlike the original Dutch (Brinkhof et al., 2022) and Portuguese (Miguel and Pedroso-Chaparro, 2025) validations—which utilized large, randomly divided samples to perform exploratory and confirmatory factor analyses on independent groups—this study lacked a second group for confirmatory analysis. Consequently, future research should prioritize using larger, more representative samples to conduct confirmatory factor analyses that further substantiate our preliminary psychometric results. Additionally, the low participation of male respondents poses a significant challenge for subgroup analysis. The resulting gender imbalance could lead to insufficient sample sizes for the stable estimation of measurement invariance. Consequently, the current lack of a balanced distribution could preclude robust comparisons between men and women, future research with larger and more heterogeneous samples is required to assess whether the scale's structural properties remain invariable across genders in the Spanish context. Additionally, it is important to note the relatively low reliability observed for the Attitudes Toward One's Own Aging Subscale (Liang and Bollen, 1983). Burton et al. (2021) conducted a systematic review, reporting that Cronbach alpha for this instrument tipically ranged between 0.61 and 0.75. This could be due to the short number of items, what could severely interfere with Cronbach's alpha. Therefore, the subscale's performance aligns with previously reported literature. Nonetheless, associations involving self-perceptions of aging reported in this manuscript should be interpreted with caution, taking into consideration these measurement limitations. Finally, the cross-sectional nature of the study does not allow causal infer-ences in the associations between the assessed variables. For example, we proposed that perceived negative ageism increases the negative perception of one's own aging. Future research with longitudinal and experimental designs is needed to understand the mechanisms of action in these associations.

Despite the above-mentioned limitations, the findings validate the utility of the Spanish version of the Perceived Ageism Questionnaire. This adaptation enables future investigations into perceived ageism with Spanish-speaking communities across various age demographics, considering both negative and positive perceived ageism. Such studies will likely clarify the underlying mechanisms and the specific link between age-based discrimination and emotional wellbeing.

## Data Availability

The raw data supporting the conclusions of this article will be made available by the authors, without undue reservation.
